# Association Between Patients’ Perceptions of the Sexual Acceptability of Contraceptive Methods and Continued Use Over Time

**DOI:** 10.1001/jamainternmed.2021.1439

**Published:** 2021-04-26

**Authors:** Jenny A. Higgins, Renee D. Kramer, Bethany Everett, Kelsey Q. Wright, David K. Turok, Jessica N. Sanders

**Affiliations:** 1 Department of Obstetrics and Gynecology, University of Wisconsin-Madison, Madison; 2 Collaborative for Reproductive Equity, University of Wisconsin-Madison, Medical Sciences Center 4245, Madison; 3 Department of Gender and Women’s Studies, University of Wisconsin-Madison, Madison; 4 Department of Population Health Sciences, University of Wisconsin-Madison, Madison; 5 Department of Sociology, University of Utah, Salt Lake City; 6 Department of Sociology, University of Wisconsin-Madison, Madison; 7 Department of Obstetrics and Gynecology, University of Utah, Salt Lake City

## Abstract

This cohort study examines the association of sexual function, satisfaction, and self-reported sexual acceptability with continued contraceptive use.

Despite contraception’s health and social benefits, many women^1^ report nonuse and discontinuation due to method dissatisfaction. Burgeoning research suggests that sexual acceptability influences contraceptive practices.^1^ In a large cohort of new-start contraceptive users, we examined the association of sexual function, satisfaction, and self-reported sexual acceptability with continued contraceptive use over time.

## Methods

Data were obtained from the HER Salt Lake Contraceptive Initiative, a cohort study (Contraceptive Trial Registration Number NCT02734199) approved by the University of Utah institutional review board.^2^ All interested participants completed the informed consent process and the baseline survey in a private room. From March 2016 to March 2017, family planning clients received their desired contraceptive method at no cost and could switch or discontinue at any time. At baseline, 1, 3, and 6 months, participants completed sexual acceptability measures including the Female Sexual Function Index (FSFI-6)^3^ and the 20-item New Sexual Satisfaction Scale (NSSS).^4^ In follow-up surveys, they reported whether, and how, their contraceptive method had affected their sex life in the past month (from “a lot worse” to “a lot better”).^5^

Investigators used multivariable logistic regression models to assess patterns of noncontinuation (switching or discontinuation) of enrollment method by 6 months. Independent variables were perceived sexual effect of method and changes in FSFI-6 and NSSS scores. Covariates included bleeding changes and frequency of adverse effects captured by the Menstrual Symptom Questionnaire, divided into physical (eg, breast tenderness) and mood-related (eg, depression) changes.^6^ Controls included enrollment method and sociodemographic factors related to contraceptive practices (eg, age, relationship status, and education). Participants selected their race(s) and ethnicity from 7 listed categories, including a write-in option.

## Results

Among 2027 eligible participants included in the analyses, 610 (30.1%) selected the levonorgestrel, 52 mg, intrauterine device (IUD), 454 (22.4%) the etonogestrel contraceptive implant, 367 (18.1%) oral contraceptive pills, 303 (15.0%) copper T380A IUD, 190 (9.4%) depo medroxyprogesterone acetate injection, and 103 (5.1%) vaginal ring. Less than 1% selected all other methods.

Participants reported no significant changes in FSFI-6 and NSSS scores (Table 1). However, at 1 month, 107 participants (52.8%) in total said their new method improved their sex life (529 [26.1%] “improved a lot”; 542 [26.7%] “improved a little”); 340 (16.8%) in total said it made their sex life worse (291 [14.4%] “a little worse”; 49 [2.4%] “a lot worse”); and 616 (30.4%) reported no sexual effect. Respondents whose method made their sex life “a lot worse” had 3.3 increased odds of noncontinuation by 6 months relative to those whose method improved their sex life “a lot” (95% CI, 1.5-7.1; *P* = .002; those reporting “a little worse” had 2.0 increased odds (95% CI, 1.3-3.0, *P* = .002. Although bleeding changes were also significantly associated with noncontinuation, changes in FSFI-6 scores, NSSS scores, and adverse effects were not (Table 2).

**Table 1.** Sexuality Measures, Bleeding Changes, and Adverse Effects, 0 to 1 Month, Among New-Start Contraceptive Users (N = 2027)

Variable	Mean (SD)
Effect of method on sex life, measured at 1 mo only, No. (%)	
Has made my sex life a lot worse	49 (2.4)
Has made my sex life a little worse	291 (14.4)
Has had no effect on my sex life	616 (30.4)
Improved my sex life a little	542 (26.7)
Improved my sex life a lot	529 (26.1)
New sexual satisfaction scale, range 20-100	
Average baseline score	75.8 (16.6)
Average change in score from 0-1 mo	−2.1 (16.9)
Female Sexual Functioning Index-6, range 5-30	
Average baseline score	23.4 (4.8)
Average change in score from 0-1 mo	−0.38 (5.3)
Changes in vaginal bleeding, measured at 1 mo only, No. (%)	
I’ve had no vaginal bleeding	297 (14.7)
I’ve had less bleeding than before	531 (26.2)
I’ve had no change from before	275 (13.6)
I’ve had more bleeding than before	924 (45.6)
Menstrual Symptoms Questionnaire: mood symptoms, range 0-5^a^	
Average baseline score	1.7 (1.3)
Average change in score from 0-1 mo	0.30 (1.5)
Menstrual Symptoms Questionnaire: physical symptoms, range 0-5^b^	
Average baseline score	1.2 (0.8)
Average change in score from 0-1 mo	0.25 (0.9)

^a^ Mood adverse effects include feelings of depression or changes in mood.

^b^ Physical adverse effects include headaches, bloating, cramping, diarrhea or constipation, acne, weight gain or loss, and breast tenderness.

**Table 2.** Unadjusted and Adjusted Multivariable Regression Results: Effects of Sexuality Measures, Bleeding Changes, and Adverse Effects on Contraceptive Noncontinuation at 6 Months (Baseline N = 2027)^a^

Variable	Noncontinuation at 6 mo [continuation is reference category]			
	Unadjusted OR (95% CI)	*P* value	Adjusted OR (95% CI)	*P* value
Effect of contraceptive method on sex life at 1 mo				
Improved my sex life a lot	1 [Reference]		1 [Reference]	
Has made my sex life a lot worse	2.97 (1.52-5.77)	.001	3.29 (1.55-7.01)	.002
Has made my sex life a little worse	1.52 (1.04-2.23)	.03	1.99 (1.28-3.10)	.002
Has had no effect on my sex life	1.50 (1.09-2.07)	.01	1.39 (0.98-1.98)	.07
Improved my sex life a little	1.05 (0.74-1.49)	.77	1.11 (0.77-1.61)	.57
Change in FSFI from 0-1 mo	1.00 (0.98-1.02)	.86	1.03 (0.99-1.06)	.11
Change in NSSS from 0-1 mo	1.00 (0.99-1.01)	.68	1.00 (0.99-1.01)	.78
Changes in bleeding reported at 1 mo				
I’ve had less vaginal bleeding than before	1 [Reference]		1 [Reference]	
I’ve had no vaginal bleeding	1.52 (1.05-2.19)	.03	1.27 (0.85-1.92)	.25
I’ve had no change in vaginal bleeding	1.82 (1.26-2.63)	.001	1.53 (1.03-2.28)	.04
I’ve had more vaginal bleeding than before	0.92 (0.68-1.24)	.58	1.15 (0.82-1.62)	.42
Change in mood adverse effects from 0-1 mo^b^	1.10 (1.01-1.19)	.02	1.04 (0.93-1.15)	.51
Change in physical adverse effects from 0-1 mo^c^	1.12 (0.98-1.29)	.09	1.15 (0.96-1.37)	.12

Abbreviations: FSFI, Female Sexual Function Index; NSSS, New Sexual Satisfaction Scale; OR, odds ratio.

^a^ Most contraceptive patients identify as women, and contraception is associated with women as a sociocultural and political group and not merely people who can get pregnant. However, we underscore that not all people who seek or use contraception identify as women. Unadjusted models include only design-related variables (site of enrollment in study, period of enrollment in study, and average length between survey responses in days). Adjusted models include these variables in addition to age, race/ethnicity, relationship status, federal poverty level category, contraceptive method, sexual orientation, and general well-being, and the other variables in the table.

^b^ Mood adverse effects include feelings of depression or changes in mood.

^c^ Physical adverse effects included headaches, bloating, cramping, diarrhea or constipation, acne, weight gain or loss, and breast tenderness.

## Discussion

Contraceptive users’ perceptions that their method negatively affected their sex life were strongly associated with noncontinuation by 6 months—more robustly than other adverse effects. Qualitative research on contraceptive sexual acceptability documents multiple domains that may help explain this effect, including sexual spontaneity, psychological disinhibition, and partner connection when feeling well protected against unwanted pregnancy.^1^

Study strengths include its large sample size, prospective design, and use of multiple measures to assess sexual experiences. Limitations of this study include that the outcome measure was affected by the large proportion of participants who initiated IUDs or implants—methods that require more effort to discontinue than pills, rings, and injections. Though we included only participants with complete variables, sensitivity analyses indicated that results held when we included all participants.

These findings suggest that the sexual acceptability of a contraceptive method may have an important role in whether a patient continues to use it. Finding methods that favorably align with users’ sexual experiences may reduce noncontinuation and improve patients’ sexual lives and well-being.
